# Extranodal Diffuse Large B-Cell Lymphoma of Bone and Soft Tissue Presenting With Marked Lymphedema and Hypercalcemia

**DOI:** 10.7759/cureus.22025

**Published:** 2022-02-08

**Authors:** Arafat Shabbir, Arsenije Kojadinovic, Sanaz Gidfar, Prabhjot S Mundi

**Affiliations:** 1 Division of Hospital Medicine, Internal Medicine, Yale New Haven Hospital, New Haven, USA; 2 Internal Medicine, Mount Sinai Medical Center, New York, USA; 3 Division of Hospital Medicine, Internal Medicine, Guthrie Corning Hospital, Sayre, USA; 4 Division of Hematology/Oncology, Internal Medicine, Columbia University Vagelos College of Physicians and Surgeons, New York, USA

**Keywords:** soft tissue lymphoma, extranodal lymphomas, hepatitis-c infection, agent orange exposure, diffuse large b cell lymphoma (dlbcl)

## Abstract

Extranodal involvement is more prevalent in diffuse large B-cell lymphoma (DLBCL) compared to other non-Hodgkin lymphoma subtypes, with up to 40% of patients with early-stage disease having at least one extranodal site. Virtually any tissue can be involved, but primary skeletal muscle and bone DLBCL is exceedingly rare. Here we report a case of DLBCL of the humerus and proximal limb musculature in a Vietnam War combat veteran with significant Agent Orange exposure and untreated hepatitis C infection. The patient presented with 1,25-dihydroxyvitamin D3-mediated malignant hypercalcemia and massive soft tissue infiltration. He had an excellent treatment response to chemotherapy and involved field radiation therapy. Also, we discuss hepatitis C and Agent Orange in the context of the pathogenesis and management of DLBCL.

## Introduction

Diffuse large B-cell lymphoma (DLBCL) accounts for 25% of all non-Hodgkin lymphomas (NHLs), with an annual incidence of 7 cases per 100,000 in the United States [1]. Most DLBCL cases are diagnosed de novo, although the condition may also arise through the transformation of more indolent forms of B-cell lymphoma [2]. While there has been an intense focus over the past two decades on the molecular classification of DLBCL into subgroups to predict therapeutic outcomes, there has also been considerable heterogeneity in its clinical presentation [3].

Extranodal involvement is more prevalent in DLBCL compared to other NHL subtypes. About 40% of patients with Ann Arbor stage I/II disease have involvement of one or more extranodal sites at presentation, although it is not usually the primary focus of disease [4]. The most common extranodal site is the gastrointestinal tract, but virtually any tissue can be involved [5]. Primary skeletal muscle and bone DLBCL is extraordinarily rare. Here we report a case of DLBCL of the humerus and proximal limb musculature in a veteran with significant Agent Orange exposure.

## Case presentation

**Figure 1:**
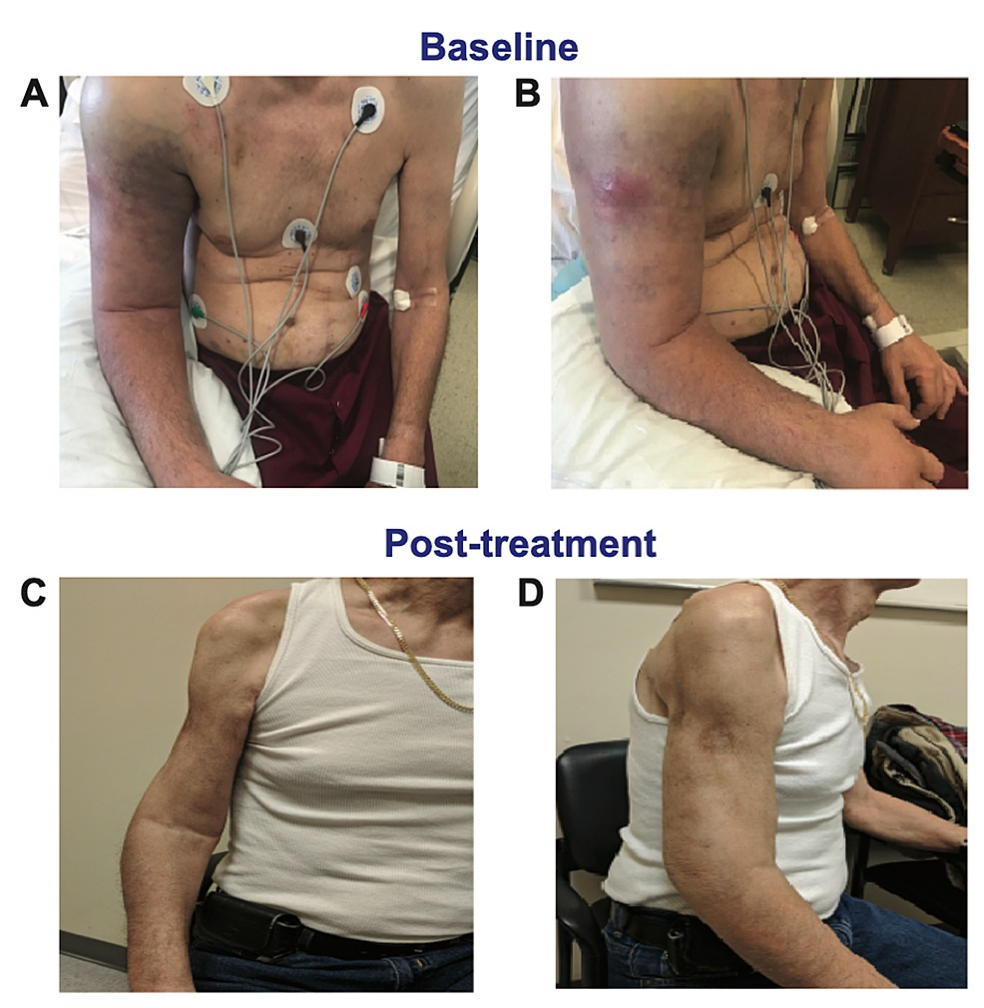
Initial and post-treatment clinical images (A) Initial presentation (anterior view). The patient presented with extensive soft tissue enlargement of the proximal right upper extremity extending to the rotator cuff muscles and surrounding edema extending down to the hand. (B) Initial presentation (lateral view). (C) Six months post-completion of chemotherapy and radiation (anterior view). There is evidence of atrophy of the triceps and biceps muscles, but the patient demonstrates adequate range of motion at the shoulder and is able to write and carry light objects. (D) Six months post-completion of chemotherapy and radiation (lateral view).

A 71-year-old male veteran who served in the Vietnam War, with an extensive history of tobacco and substance use in remission and untreated hepatitis C virus (HCV) infection, presented to his primary care physician with several weeks of progressive, extensive non-traumatic lymphedema of the right upper extremity (RUE). Two duplex ultrasounds were negative for thrombosis of the right brachial, axillary, and subclavian deep veins. He was referred to the vascular surgery clinic and trained on the use of a lymphedema pump, but subsequently presented to the emergency department one week later with severe pain and worsening edema (Figures 1A, 1B). Routine laboratory studies were notable for marked hypercalcemia to 13.5 mg/dL (normal: 8.6-10.3) with normal albumin (3.7 g/dL) and phosphorus levels (3.1 mg/dL). The patient was admitted to the hospital for further management and a workup for hypercalcemia was initiated.

Over the next 72 hours, the patient received continuous intravenous hydration with normal saline and several doses of calcitonin, with minimal improvement in the calcium level to 12.9 mg/dL. The parathyroid hormone (PTH) level was appropriately suppressed to 6.8 pg/mL (normal: 10-65), 24-hour urinary calcium excretion was increased to 488 mg, PTH-related peptide was not detected, and the 1,25-dihydroxyvitamin D3 level was 160 pg/mL (normal: 19.9-79.3). The serum lactate dehydrogenase level was 3471 units/L (normal: 94-260). The principal differential diagnosis was sarcoidosis or lymphoma.

**Figure 2:**
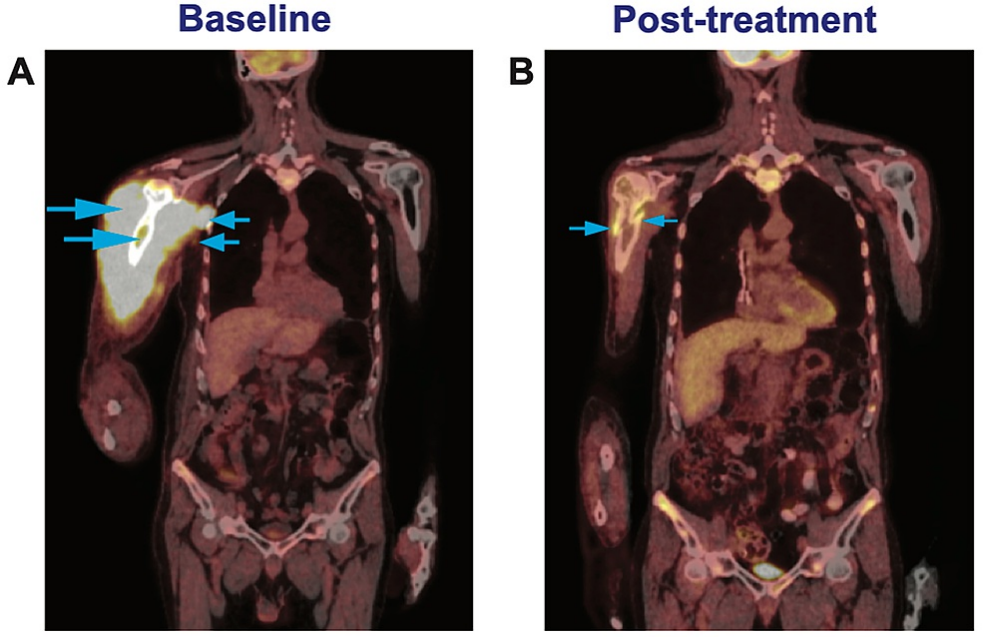
PET scan images (A) Initial staging PET scan. There is marked FDG avidity of the right humerus, RUE proximal musculature, and right rotator cuff muscles and at least two hypermetabolic right axillary lymph nodes (arrows). There is an absence of significant metabolic activity in any of the other major lymph node groups, bones, muscles, or visceral organs. (B) Six months post-treatment PET scan. There is evidence of complete metabolic response with an exception of the disjoint edges of cortical bone (arrows) along a remnant pathological fracture of the right humerus. FDG: 18-fluorodeoxyglucose; PET: positron emission tomography; RUE: right upper extremity

A computed tomography (CT) scan demonstrated a few sub-centimeter calcified pulmonary nodules with the appearance of chronic granulomas, but no evidence of hilar or mediastinal lymphadenopathy, significantly reducing the likelihood of sarcoidosis. A CT scan of the RUE demonstrated cortical destruction of the humeral head and proximal shaft, along with infiltrative enlargement of the muscles of the proximal RUE and marked soft tissue edema. There were also a few enlarged axillary lymph nodes, the largest measuring 2 cm in short-axis. A positron emission tomography (PET) scan demonstrated striking 18-fluorodeoxyglucose (FDG) avidity in the humerus and surrounding musculature (maximum standardized uptake value [mSUV] 10-15), as well as two FDG-avid axillary lymph nodes with mSUV 5 and 14 (Figure 2A). No other hypermetabolic lymphadenopathy was present.

The patient was started on prednisone 20 mg daily and there was rapid normalization of the calcium level to 9.9 mg/dL within 48 hours of initiation. An excisional lymph node biopsy was performed. Histopathologic evaluation demonstrated several large neoplastic cells with effacement of the normal lymph node architecture. Flow cytometry and immunohistochemistry revealed a neoplastic cell population positive for CD45, CD19, CD20, CD79 and Bcl-2 and negative for CD5, CD10, and pancytokeratin (AE1/AE3). About 80% of tumor cells expressed Bcl-6 and 10% were positive for MUM1. The Ki-67 proliferative index was 60%. The findings on imaging and pathology were consistent with stage IE (one contiguous extralymphatic site) DLBCL. Risk stratification by the revised International Prognostic Index placed the patient into the intermediate risk group (2 points: age >60, elevated lactate dehydrogenase [LDH]), with predicted four-year disease-free survival of 70%-80% with standard chemotherapy. While the expression of MUM1 was <30%, staining can be subject to sampling bias, and the overall pattern suggested the possibility of the unfavorable *activated B-cell phenotype* (ABC type).

In spite of advances in our understanding of DLBCL pathobiology, R-CHOP (rituximab, cyclophosphamide, doxorubicin, vincristine, and prednisone) remains the standard-of-care first-line regimen for both *germinal center B-cell like* (GCB) and ABC types. Our patient was administered three cycles of R-CHOP and underwent an interval PET scan that demonstrated residual metabolic activity in the proximal humerus and surrounding soft tissue, albeit with a dramatic reduction in mSUV (2-5 vs. 10-15), and he reported partial recovery of RUE movement. Thus, a decision was made to proceed to six cycles of R-CHOP followed by involved field radiation therapy of 30 Gy. Following the completion of DLBCL-directed therapy, he was evaluated in the infectious disease clinic, and completed eight weeks of anti-HCV treatment with glecaprevir/pibrentasvir, resulting in sustained virologic response. The patient is now in remission for more than 30 months, with marked improvement in RUE function (Figures 1C, 1D) in spite of residual pathological fracture in the humerus with cortical non-healing (Figure 2B).

## Discussion

We report an exceptionally uncommon presentation of DLBCL with predominant involvement of bone and skeletal muscle in a veteran with Agent Orange exposure and chronic HCV infection. Bone involvement represents only 3%-5% of extranodal DLBCL, while skeletal muscle involvement is exceedingly rare, accounting for less than 0.5% of cases [5-7]. Lymphoma associated hypercalcemia can initially be effectively controlled with glucocorticoids, which effectively inhibits gastrointestinal absorption of calcium, including through inhibition of 1-alpha-hydroxylase [8,9]. Chronic viral infections that result in immune depletion or dysregulation, such as HIV, are strongly associated with NHL incidence and directly linked to its pathogenesis. HCV is associated with only a modest two- to threefold relative risk of B-cell NHL, most commonly splenic marginal zone lymphoma, as well as de novo or transformed DLBCL, and less commonly follicular lymphoma [10,11].

Nonetheless, there is some evidence to suggest a causal relationship between HCV and lymphoma, and direct antiviral agents alone may lead to remission in some indolent cases [10,12]. The presence of specific recurrent immunoglobulin rearrangements in transformed cells supports the hypothesis that sustained antigenic stimulation leads from oligoclonal to monoclonal expansion of lymphocyte populations that will sometimes progress to malignancy [13]. There is also evidence that HCV can enter lymphocytes and thus may play a direct role in lymphomagenesis [14].

Furthermore, successful eradication of HCV may improve long-term outcomes in DLBCL. In a study by Pellicelli et al., a cohort of 21 patients with HCV and DLBCL received antiviral therapy during or shortly after achieving remission with chemotherapy [15]. Relapse was uncommon when HCV eradication was successful, but DLBCL relapse occurred in four out of five patients without sustained virologic response. Hosry et al. reported on a retrospective nested case control study [16]. Patients with untreated HCV and DLBCL demonstrated significantly lower complete response rates to first-line chemotherapy compared to HCV-negative patients (66% vs. 83%) and had significantly worse five-year overall survival (hazard ratio 2.3; 95% CI: 1.01-5.30). Together, these studies provide impetus for the prompt treatment of HCV in patients diagnosed with DLBCL in the era of highly effective direct antiviral agents.

Agent Orange and non-Hodgkin lymphoma

Various phenoxy herbicides were used during the conflict in the Indo-Chinese peninsula from 1962 to 1971, principally as defoliants to allow opposition movement to be monitored from air [17,18]. Of these, Agent Orange was most abundant with over 12 million gallons sprayed between 1965 and 1970. A controversial body of epidemiologic evidence links Agent Orange exposure to neurodegenerative conditions and multiple forms of cancer, including NHL and soft tissue sarcomas. These conditions emerge several decades after exposure.

Agent Orange is readily contaminated with congeners during its manufacture including the potent carcinogen 2,3,7,8-tetrachlorodibenzo-ρ-dioxin, or TCDD [19]. Dioxins are lipophilic, chlorinated aromatic hydrocarbons that are rapidly absorbed through the skin and distributed throughout the body. The median half-life for clearance is seven years but varies considerably between individuals. TCDD binds and activates the aryl hydrocarbon receptor (AHR), a ligand-activated transcription factor that is expressed in almost all tissues in the body and plays a complex role in regulating many genes involved in immunity, stem cell maintenance, and cellular differentiation, as well as cytochrome P450 genes involved in drug metabolism/conjugation [20]. Three million American service members were exposed via direct handling during the Air Force's Operation Ranch Hand or through ground combat in defoliated regions.

Several studies suggest at least a modest association between Agent Orange exposure and NHL mortality, including studies with matched veteran controls deployed in areas without spraying. The Selected Cancers Study showed that Vietnam service was associated with a 47% increased incidence of NHL (odds ratio 1.47; 95% CI: 1.1-2.0) [21]. However, the lack of a clear exposure-risk gradient, the overall modest effect, the long latency period and the unknown long-term mechanism of carcinogenesis have made it difficult to establish causality [22-24]. Furthermore, available studies provide little information about the incidence of specific NHL subtypes; it is clear that some risk factors are associated with specific subtypes, e.g., *Helicobacter pylori* and gastric marginal zone lymphoma. The route of exposure to Agent Orange, predominantly through direct cutaneous absorption or ingestion of contaminated food, may also be important.

Our patient described prolonged periods of crawling on all fours while engaged in ground combat during his close to two years of service. The predilection of dioxins to be absorbed through and accumulate in soft tissue and the unique association of Agent Orange and other phenoxy herbicides with NHL and soft tissue sarcomas imply a possible pathobiologic basis in his development of extranodal DLBCL.

## Conclusions

While extranodal involvement is more prevalent in DLBCL relative to other lymphoma types, primary skeletal muscle and bone DLBCL is exceedingly rare. In patients diagnosed with DLBCL who also have active HCV infection, successful eradication of HCV may improve long-term outcomes in DLBCL. An accumulating and controversial body of epidemiologic evidence links Agent Orange exposure to increased incidence of multiple forms of cancer, including lymphoma.
